# Development of a theory-informed implementation intervention to improve the triage, treatment and transfer of stroke patients in emergency departments using the Theoretical Domains Framework (TDF): the T^3^ Trial

**DOI:** 10.1186/s13012-017-0616-6

**Published:** 2017-07-17

**Authors:** Louise E. Craig, Natalie Taylor, Rohan Grimley, Dominique A. Cadilhac, Elizabeth McInnes, Rosemary Phillips, Simeon Dale, Denise O’Connor, Chris Levi, Mark Fitzgerald, Julie Considine, Jeremy M. Grimshaw, Richard Gerraty, N. Wah Cheung, Jeanette Ward, Sandy Middleton

**Affiliations:** 1 Nursing Research Institute, St Vincent’s Health Australia (Sydney) and Australian Catholic University, Executive Suite, Level 5 deLacy Building, St Vincent’s Hospital, 390 Victoria Street, Darlinghurst 2010, New South Wales, Australia; 2 Cancer Research Division, Cancer Council NSW, 153 Dowling St, Woolloomooloo, NSW 2011 Australia; 3 Sunshine Coast Hospital and Health Service/Sunshine Coast Clinical School, The University of Queensland, Nambour, QLD Australia; 4 Stroke and Ageing Research, School of Clinical Sciences at Monash Health, Monash University, Clayton, Victoria Australia; 5 Florey Institute of Neuroscience and Mental Health, University of Melbourne, Parkville, Victoria Australia; 6 School of Public Health and Preventive Medicine, Monash University, Level 1, 549 St Kilda Road, Melbourne, VIC Australia; 7 John Hunter Hospital, Newcastle, Australia; 8 Centre for Translational Neuroscience and Mental Health, University of Newcastle/Hunter Medical Research Institute, Newcastle, Australia; 9 Alfred Hospital, Melbourne, Victoria 3004 Australia; 10 Department of Surgery, Central Clinical School, Monash University, Melbourne, Australia; 11 National Trauma Research Institute, Melbourne, Australia; 12 School of Nursing and Midwifery and Centre for Quality and Patient Safety Research – Eastern Health Partnership, Deakin University, Geelong, Victoria 3220 Australia; 13 Clinical Epidemiology Program, Ottawa Health Research Institute, 1053 Carling Avenue, Administration Building, Room 2-017, Ottawa, Ontario K1Y 4E9 Canada; 14 Department of Medicine, University of Ottawa, 451 Smyth Road, Ottawa, Ontario K1H 8M5 Canada; 15 Department of Medicine, Monash University, Neurosciences Clinical Institute, Epworth hospital, Richmond, Victoria 3121 Australia; 16 Centre for Diabetes and Endocrinology Research, Westmead Hospital and University of Sydney, Westmead, Sydney, NSW Australia; 17 School of Epidemiology, Public Health and Preventive Medicine (SEPHPM), University of Ottawa, 451 Smyth Road, Ottawa, Ontario K1H 8M5 Canada; 18 Nulungu Research Institute, University of Notre Dame Australia, Broome, Western Australia Australia

**Keywords:** Implementation intervention, Theoretical Domains Framework, Behaviour change techniques

## Abstract

**Background:**

Theoretical frameworks and models based on behaviour change theories are increasingly used in the development of implementation interventions. Development of an implementation intervention is often based on the available evidence base and practical issues, i.e. feasibility and acceptability. The aim of this study was to describe the development of an implementation intervention for the T^3^ Trial (**T**riage, **T**reatment and **T**ransfer of patients with stroke in emergency departments (EDs)) using theory to recommend behaviour change techniques (BCTs) and drawing on the research evidence base and practical issues of feasibility and acceptability.

**Methods:**

A stepped method for developing complex interventions based on theory, evidence and practical issues was adapted using the following steps: (1) Who needs to do what, differently? (2) Using a theoretical framework, which barriers and enablers need to be addressed? (3) Which intervention components (behaviour change techniques and mode(s) of delivery) could overcome the modifiable barriers and enhance the enablers? A researcher panel was convened to review the list of BCTs recommended for use and to identify the most feasible and acceptable techniques to adopt.

**Results:**

Seventy-six barriers were reported by hospital staff who attended the workshops (step 1: thirteen TDF domains likely to influence the implementation of the T^3^ Trial clinical intervention were identified by the researchers; step 2: the researcher panellists then selected one third of the BCTs recommended for use as appropriate for the clinical context of the ED and, using the enabler workshop data, devised enabling strategies for each of the selected BCTs; and step 3: the final implementation intervention consisted of 27 BCTs).

**Conclusions:**

The TDF was successfully applied in all steps of developing an implementation intervention for the T^3^ Trial clinical intervention. The use of researcher panel opinion was an essential part of the BCT selection process to incorporate both research evidence and expert judgment. It is recommended that this stepped approach (theory, evidence and practical issues of feasibility and acceptability) is used to develop highly reportable implementation interventions. The classifying of BCTs using recognised implementation intervention components will facilitate generalisability and sharing across different conditions and clinical settings.

**Electronic supplementary material:**

The online version of this article (doi:10.1186/s13012-017-0616-6) contains supplementary material, which is available to authorized users.

## Background

Evidence-based guideline recommendations are available for the early management of patients with acute stroke. Early diagnosis of stroke in emergency departments (EDs); administration of recombinant tissue plasminogen activator (rt-PA) and endovascular clot retrieval to eligible patients; and management of fever, hyperglycaemia and swallowing difficulties before transfer to a stroke unit are essential elements of evidence-based stroke care and recommended in current clinical guidelines [1]. Yet, inappropriate triage [2] and delays in diagnosis, treatment and transfer of stroke patients from ED to stroke units still occur [1]. The T^3^ Trial is a prospective, multicentre, parallel group, blinded, cluster randomised trial that aimed to evaluate the effectiveness of an implementation intervention to improve the triage, treatment and transfer of stroke patients from ED to stroke units on 90-day outcomes and in-hospital processes of care [3]. This paper describes the development of the theory-based implementation intervention for this Trial.

The use of theory in the intervention development process has been identified by the UK Medical Research Council (MRC) as crucial to increase intervention effectiveness by targeting causal determinants of behaviour and facilitate an understanding of what works (i.e. the mechanisms of change) [4, 5]. Several approaches have been proposed that integrate the use of theory in implementation intervention development [5–7]. Although there are studies that apply these approaches in the process of developing an implementation intervention [8–10], frequently, these interventions are still based on intuitive or non-theoretical methods [11]. There is also a lack of detailed reporting of the process of intervention development and the content of the implementation intervention which, if available, would assist replication and advance the knowledge base about the optimum approach for intervention development [12].

The Theoretical Domains Framework (TDF) is a framework of 14 theoretical domains derived from 33 behaviour change theories developed using a process of expert consensus with subsequent validation work [13, 14]. The TDF has successfully been applied in a number of healthcare settings to (i) guide intervention development for the implementation of guidelines or clinical interventions [6, 9, 15], (ii) characterise, according to theory, an existing intervention to implement evidence-based care to facilitate accurate replication [16, 17], and (iii) understand factors that may inhibit uptake of an intervention [18, 19]. The additional benefit of the TDF is that behaviour change techniques (BCTs) have been pre-assigned to each of the TDF domains [20]. Two matrices which assign the most appropriate BCTs to each of the TDF domains have previously been developed by Cane et al. [20] and by Michie et al. [5].

The implementation of complex clinical interventions, such as those that have numerous intervention components, as is the case in the T^3^ Trial, often involve the use of theory but may also require incorporation of the evidence base and consideration of practical issues such as feasibility and acceptability [21]. Firstly, theory is important to understand the factors influencing clinician behaviours and to guide the use of appropriate behavioural change techniques (BCT), the smallest components of an implementation intervention [6]. Secondly, evidence regarding technique effectiveness can assist the selection of BCTs and the best mode of delivery [6]. This might be generic behaviour change evidence but also might incorporate context-specific evidence, from the stroke or ED literature in the case of this study. Thirdly, an understanding of practical issues (feasibility and acceptability) and expert clinical judgment can guide the selection of the most relevant BCTs for a particular context [6]. Some studies have incorporated stakeholder opinion in the design of implementation interventions to incorporate practical considerations and judgment [6, 9, 10, 22]. As contextual issues have a significant influence on the delivery and impact of complex clinical interventions [4], a theory-based evidence-driven approach which takes into account context should be considered in developing implementation interventions. However, there are very few well reported studies that use this stepped approach of intervention development (theory, evidence and practical issues of feasibility and acceptability).

The aim of this study was to describe the development of an implementation intervention (i) using theory to inform selection of BCTs for the T^3^ Trial, (ii) further guided by the evidence of effectiveness of implementation interventions (including that from a previous acute stroke implementation trial [23]) and (iii) consideration of researcher opinion to select appropriate BCTs. This implementation intervention will be subsequently tested under trial conditions [3].

## Methods

The T^3^ Trial clinical intervention is an evidence-based care bundle of clinical protocols for triage, treatment and transfer of patients following acute stroke and comprised of 12 different clinical care elements (hereonin referred to as ‘target behaviours’ [Table 1]) [3]. As the T^3^ trial clinical intervention consisted of ‘a number of separate elements which seem essential to the proper functioning of the intervention although the ‘active ingredient’ of the intervention that is effective is difficult to specify’, it meets the MRC definition of a complex intervention [4]. Four of the five-stepped method proposed by French et al. [6] for developing complex interventions based on theory, evidence and practical issues were undertaken as follows:

**Table 1:** Target clinical behaviours for T^3^ trial

Target behaviour	Target clinical behaviour (includes timepoint if not immediate) Location: emergency department	Who performs the behaviour
Triage	All patients presenting to ED with signs and symptoms of suspected acute stroke should be triaged as Australian Triage Scale Category 1 or 2 (seen within 10 min)	ED nurse
Thrombolysis	All patients to be assessed for rt-PA eligibility in ED All eligible patients to receive rt-PA in ED	ED nurse, ED doctor, Stroke doctor, Stroke nurse ED doctor, Stroke doctor, Stroke nurse
Temperature management	All patients to have their temperature taken on admission to ED and then at least 4 hourly whilst they remain in ED Temperature 37.5 °C or greater to be treated with paracetamol (acetaminophen) in ED	ED nurse ED nurse
Blood glucose management	Venous BGL sample taken to laboratory on admission to ED Finger prick BGL recorded on admission to ED and finger prick BGL monitored every 6 h (or greater if elevated) Insulin administered to all patients with BGL > 10 mMol/L within 1 h in ED or stroke unit	ED nurse, ED doctor ED nurse, Stroke nurse ED nurse, Stroke nurse, Endocrinologist
Swallow management	Patients to remain NBM until a swallow screen by non-Speech pathologist or swallow assessment by Speech pathologist performed in ED All patients who fail the swallow screen to remain NBM and have a swallowing assessment by a Speech pathologist whilst in ED	ED nurse, Stroke nurse, ED doctor, Speech pathologist Speech pathologist
Transfer	All patients with stroke to be discharged from ED within 4 h All patients with stroke to be admitted to the hospital’s stroke unit	ED nurse, ED doctor, Stroke nurse, Bed manager ED nurse, Stroke nurse, Bed manager

*BGL* blood glucose level, *ED* emergency department, *NBM* Nil by mouth, *rt-PA* recombinant tissue plasminogen activator

- Step 1: Who needs to do what, differently?
- Step 2: Using a theoretical framework, which barriers and enablers need to be addressed?
- Step 3: Which intervention components (behaviour change techniques and mode(s) of delivery) could overcome the modifiable barriers and enhance the enablers?

Step 4: ‘How can behaviour change be measured and understood?’ previously has been reported in our published protocol paper (primary and secondary outcomes with an a priori planned process evaluation) [3]. Step 5: ‘How can behaviour change be sustained’ is beyond the scope of the T^3^ Trial.

### Step 1: Who needs to do what, differently?

Twelve evidence-based targeted behaviours were identified by the trial investigators for the triage, treatment and transfer (T^3^) elements of the intervention. We selected the target clinical behaviours to be addressed, based on documented evidence-practice gaps. As per French et al.’s approach we specified the target behaviours in detail by asking the following questions: What is the clinical behaviour that you will try to change? Who performs the behaviour(s)? And when and where do they perform the behaviour(s)?

### Step 2: Using a theoretical framework, which barriers and enablers need to be addressed?

One barrier and enabler multidisciplinary workshop (1-h duration) was conducted at each of the thirteen T^3^ Trial intervention hospitals across three Australian states and the Australian Capital Territory between October 2014 and December 2014. Purposive sampling was used to select workshop participants who could provide detailed feedback on barriers and enablers to the T^3^ Trial clinical intervention, namely (i) senior healthcare professionals working in ED (e.g. emergency physician, emergency nurses) or in stroke units (e.g. stroke physician, stroke nurses, endocrinologists, speech pathologists and bed managers) and (ii) involved in routine delivery of the target behaviours. The workshops were aimed at identifying the perceived barriers and enablers that may influence the uptake of each of the target behaviours. The workshops were facilitated by SD and SM with assistance from emergency, neurology and endocrine physician T^3^ Trial researchers. A standard presentation was given at the workshops to provide consistent information about each of the target behaviours. The workshop participants were asked to nominate specific barriers for each of the behaviours and specific enablers and strategies that could be used to overcome the barriers.

The workshops were audio recorded and transcribed verbatim. The interview transcripts were coded using thematic analysis by a single coder (LC) according to the TDF domains [14]. Individual barriers were classified to the relevant domain of the TDF. The constructs, that is the concepts provided for each of the TDF domains, were used to assist interpretation and to ensure accurate assignment of the TDF domains. The coding framework was devised by the lead author (LC) and RP applied this framework to a subset of transcripts (*n* = 5) to test the interpretation of the codes. A third researcher (NT), with expertise in the application of the TDF to primary data, independently checked the assignment of all transcript data to the TDF domains. It was agreed that should a number of barriers be reflected by more than one TDF domain, the most relevant domain should be selected. Discrepancies in allocation were resolved by reviewing and discussing the transcript dialogue before and after the extract. The TDF constructs and contextual information reported for an individual barrier/enabler were also used to understand and resolve any discrepancies.

### Step 3: Which intervention components (behaviour change techniques and mode(s) of delivery) could overcome the modifiable barriers and enhance the enablers?

The Cane et al. matrix [20] which recommended the most appropriate BCTs to each of the TDF domains was primarily used to identify BCTs. Two domains (memory, attention and decision processes and social/professional role and identity) do not have any specified BCTs in the Cane et al. matrix; therefore, a similar matrix previously developed by Michie et al. was used to inform the BCTs for these domains and other domains where the recommended BCTs were considered more appropriate than that recommended by the Cane et al. matrix. The BCT taxonomy [24] was also provided to the researchers as a resource to use where neither of the two matrices identified an appropriate BCT. Whilst these tools are useful for assigning relevant BCTs, they do not incorporate the evidence regarding implementation intervention effectiveness or issues of feasibility or acceptability. To address this, we used a pragmatic approach to selecting BCTs using the T^3^ Trial investigators knowledge of the clinical intervention and experience of the clinical context. A panel of five T^3^ Trial investigators and researchers (SM, DC, RG [a stroke physician], RP and ES), all of which had experience of applying the TDF in stroke implementation research, independently reviewed the list of matrix-assigned BCTs and from this identified the most feasible and acceptable techniques for the T^3^ Trial.

It was identified that the selection of BCTs for a single behaviour would be time consuming likely taking up to two and half hours to complete; therefore, due to time constraints of the researcher panellists, it was considered unfeasible for them to complete the selection process for all 12 behaviours. Instead, the panel were instructed to select BCTs to address the barriers associated with only one of the target behaviours *Administration of insulin to all patients with BGL* > *10 mMol/L within 1 hour by insulin infusion*. This behaviour was selected as it represented nearly every TDF domain (11 out of 13) mapped in step 1 which would allow the findings to then be applied to the remaining behaviours. The panel were provided with a number of resources (shown in brackets below) and specifically asked to

1. Identify the BCTs considered appropriate (feasible and acceptable to clinicians) e.g. techniques that are time efficient in an ED setting; resource 1: barrier extracts and TDF definitions [Additional file 1]; resource 2: domains with corresponding BCT and definitions [Additional file 2]; resource 3: technique definition and examples [Additional file 3]; resource 4: enabler data from the barrier and enabler workshops [Additional file 4])
2. Identify the BCTs considered inappropriate (not feasible and unacceptable to clinicians) e.g. techniques that reduce the need for clinical decision-making; resources 1 to 4
3. Identify any TDF domains where none of the BCTs identified by the primary Cane et al. matrix [20] were viewed as appropriate; resources 2 and 3
4. Identify further BCTs not selected by the primary Cane et al. matrix [20] e.g. techniques identified in other BCT matrices and taxonomies [5, 24] that were considered to be more appropriate; resources 2 and 3
5. Devise strategies to operationalise the selected BCTs; resource 5: table of evidence [9] to present different modes of delivery, e.g. face-to-face education meetings and local opinion leaders, based on Cochrane Effective Practice and Organisation of Care [EPOC] reviews (Additional file 5) [25–30]

**Fig. 1:**
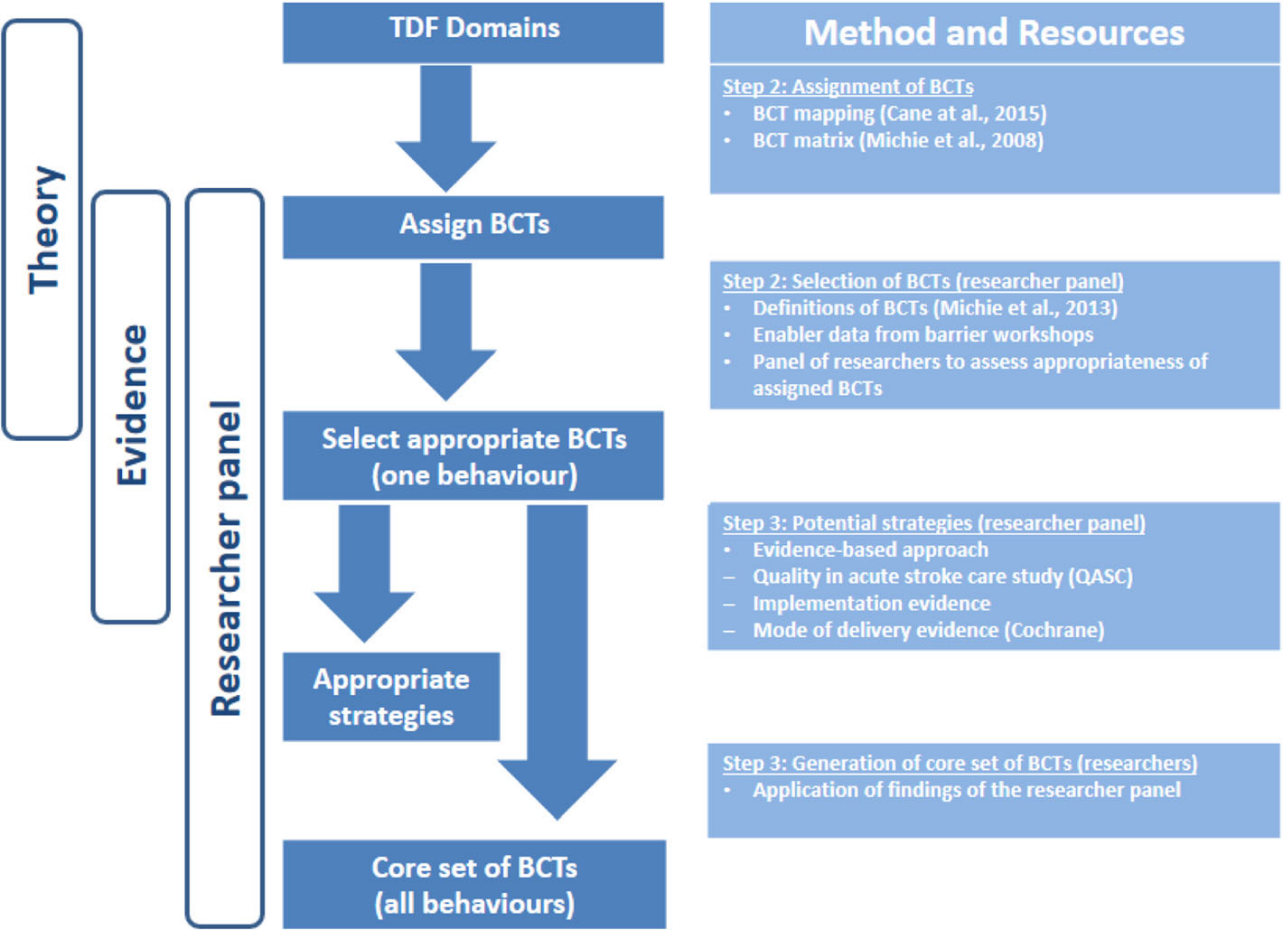
Selecting behavioural change techniques and strategies to inform the T^3^ Trial implementation intervention

In summary, each of the researchers were asked to select the most appropriate BCT based on the following criteria; feasibility of use in the ED context, personal experience of use, local relevance and acceptability. An evidence table providing effectiveness data for commonly used modes of delivery such as face-to-face education meetings and local opinion leaders was included to assist the researchers in suggesting strategies to operationalize the BCTs in an ED context. Relevant qualitative data extracts generated from the barrier and facilitator workshops were also included to allow the researchers to assess the feasibility of using the technique in ED (further details can be provided on request). An overview of this process is provided in Fig. 1. As the TDF domains mapped in step 2 were also represented in the remaining 11 behaviours, the researchers were advised that their selections would inform the final set of BCTs to be applied across all the behaviours. The researcher completed the task independently with the lead author (LC) available to guide the researchers through the process and respond to any questions to ensure adherence to the instructions provided. A BCT was included in the final set if it was selected by at least one of the researchers. The BCTs were tabulated and the frequency of selection by the researchers was reported. The selections of the researcher panel were applied by LC to the remaining 11 behaviours to create a final set of BCTs for the T^3^ Trial implementation intervention. In order to increase the transferability of the reporting of the implementation intervention, the final set of BCTs were classified by LC according to general evidence-based intervention components (BCTs and mode of delivery) commonly reported in the implementation literature [23]. These were as follows: multidisciplinary barrier and enabler workshop [31], interactive and didactic education programme [32, 33], use of opinion leaders [25], reminders [34] and site support [34]. Key relevant literature examples which presented BCTs by intervention components were used to classify accordingly [6, 9].

## Results

### Step 1: Who needs to do what, differently?

The target behaviours for the T^3^ trial intervention arose from an extensive literature review and the stroke guidelines. These targeted behaviours along with who performs the behaviour, the timepoint and the location that the behaviour occurs are listed in Table 1. These target behaviours were chosen because they had supporting evidence and were potentially modifiable at a clinician level.

### Step 2:

Thirteen workshops were conducted with 105 staff from 13 hospitals. Workshop group size ranged from minimum of five participants to maximum of 11 participants. These multidisciplinary workshops were facilitated by the researchers and included senior nurses from ED and stroke units, medical practitioners (ED physicians, neurologists, endocrinologists and their junior doctors), speech pathologists, and nurse managers. Seventy-six barriers were identified by participants (Table 2). All barriers were mapped to at least one TDF domain. For example, barriers relating to the *knowledge* domain were associated with gaps in staff’s understanding, awareness or content knowledge for certain target behaviours. This resulted in clinical uncertainty and the conduct of practices not compliant with national guidelines. Barriers relevant to the *skills* domain were related to the conduct of a specific task such as a swallow screen or a lack of skill development opportunity. Barriers assigned to the *social/professional role and identity* domain were related to professional boundaries, i.e. limited prescribing rights for nurses and professional identity, i.e. opposition to blurring of roles. Barriers relevant to the *beliefs about capabilities* domain were related to professional confidence, i.e. decision-making or low self-esteem to perform tasks related to the target behaviour. The main area of disagreement between the researchers conducting the mapping related to overlap between two domains, *beliefs about capabilities* and *social/professional role and identity.* For example, one researcher understood a barrier to conducting a clinical task to be related to a clinician’s self-confidence so mapped the extract to beliefs about capabilities domain whilst the other researcher understood the barrier to be related to professional boundaries, i.e. the task was not traditionally undertaken by that professional group so mapped the extract to social/professional role and identity. Areas of disagreement were resolved by discussion between the two researchers. No barriers were assigned to the *intentions* domain. Furthermore, the same barrier was often reported for more than one of the behaviours, for example, *competing priorities in a busy emergency department environment* was reported for four different behaviours. Overall, the same nine barriers were reported for the different target behaviours.

**Table 2:** Barriers identified for T^3^ target behaviours by Theoretical Domains Framework domain [36]

Domain and example quotes [target behaviour]	Target behaviour	Barriers identified
Knowledge (*n* ^a^ = 16) An awareness of the existence of something *They would need intensive education.* [Triage] *I think that if nurses are educated on the importance of having the temperature taken, the compliance will fit in.* [Temperature management]	Triage	Possible lack of knowledge of triaging stroke patients using the Australasian Triage Scale Delays in identifying symptoms of stroke
	Thrombolysis	Not recognising importance of documenting ineligibility for rt-PA treatment Uncertainty about use of criteria to select patients for rt-PA^b^
	Temperature management	Lack of awareness and/or do not understand importance of monitoring temperature in stroke patients Lack of knowledge about alternative modes of delivering paracetamol for patients with certain needs i.e. NBM Limited or no access to IV or rectal paracetamol for patients who are NBM Nurses reluctance to use rectal paracetamol as invasive or possibly patient refusal may result in nurse refusal to use Nurses routinely treat at a higher temperature threshold according to hospital policies
	Blood glucose management	Lack of understanding of importance of undertaking a formal BGL Lack of understanding of importance of monitoring BGL Lack of understanding of importance of administering insulin for all stroke patients regardless of diabetic status Lack of knowledge about process of administering insulin infusion Sceptism about benefits of administrating insulin for patients with a BGL > 10, e.g. risk of hypoglycemia^b^
	Swallow management	Lack of knowledge that all patients who fail swallow screen should be assessed by a speech pathologist Nurses reluctance to keep patients NBM due to lack of awareness of evidence that aspirin can be administered up to 48 hours post-stroke, i.e. may not need to be given immediately Belief of lack of robust evidence for effectiveness of non-oral aspirin when patients are NBM
Skills (*n* = 4) An ability or proficiency acquired through practice *It’s the wards, there’s a lot of wards not use to running infusions [that may be commenced in ED].* [Blood glucose management] *We struggle with the skills …we have our normal competencies, we have trouble keeping up to date with [them].* [Blood glucose management]	Triage	Possible lack of experience in triaging of stroke patients
	Temperature management	Lack of knowledge about alternative modes of delivering paracetamol for patients NBM^b^
	Blood glucose management	Lack of skill in administering an insulin infusion
	Swallow management	Lack of nurses trained how to conduct of swallow screening
Social/Professional Role and Identity (*n* = 4) A coherent set of behaviours and displayed personal qualities of an individual in a social or work setting *Oh these patients they're Category 1 or 2 [Australian Triage Scale] so there's not necessarily the need for a nurse to initiate it. You can have a physician there at the bedside as well.* [Thrombolysis] *I don’t have a problem with it, we certainly have spoken about this over the last few years, but it has been about getting support from speech pathology to roll it [nurse screening] out.* [Swallow management]	Thrombolysis	Delays associated with securing a CT scan^b^
	Temperature management	Nurses are unable to administer non-oral paracetamol without a written order^b^
	Blood glucose management	Inconsistent use or variation in protocols between between ED and stroke unit
	Swallow management	Perception that role boundaries should not be blurred, i.e. traditional discipline-specific tasks should not be conducted by staff from other disciplines.
Beliefs about Capabilities (*n* = 5) Acceptance of the truth, reality, or validity about an ability, talent, or facility that a person can put to constructive use *So I'm just wondering whether we need some more education in terms of tPA to try and make clinicians more comfortable in the use of it for strokes.* [Thrombolysis] *But the nurses having a bit more confidence to say “well no actually they haven’t had their swallow screen.”* [Swallow management]	Thrombolysis	Uncertainty about use of criteria to select patients for rt-PA^b^
	Swallow management	Nurses lack confidence to disagree with a doctor’s decision to override a patient’s NBM statu*s* ^b^ Delays in authorisation of new protocols/forms by hospital management committees^b^ Nurses’ own perception of competence in performing a swallow screen Lack confidence in performing a swallow screen
Optimism (*n* = 2) The confidence that things will happen for the best or that desired goals will be attained *The stuff that you're talking about - doing, a temperature check and the blood sugar - it's all routine stuff anyway. That's just what they [nurses] would do.* [Temperature management] *But I think getting used to just writing up for every patient with a stroke, and whether all the nurses use it.* [Temperature management]	Temperature management	Perception that this action already routine practice Attitude by nurses that changing practices about temperature management requires time
Beliefs about Consequences (*n* = 9) Acceptance of the truth, reality or validity about outcomes of a behaviour in a given situation *But with the unimpressive previous studies with stroke I don't think any of the consultants here feels that it's particularly worth pushing. I mean if it's [BGL] above 12 then we probably would do something.* [Blood glucose management] *I think you're right there is a fear of hypoglycaemia, especially in stroke patients who are obviously a slightly different group who may be NBM [and] not be getting any feeding at all. So [with] a BSL of 10.1 and then putting them on insulin infusion when they're not eating anything starts to become also a little bit of a concern.* [Blood glucose management]	Triage	Lack of understanding regarding importance of triaging stroke patients Belief that triage allocation will not impact on the patient’s outcome
	Temperature management	Lack of awareness of the importance of monitoring temperature in stroke patients Nurses reluctance to use rectal paracetamol as invasive or possibly patients may refuse may result in staff reluctance to use
	Blood glucose management	Belief that introducing insulin infusions will have unintended consequences i.e. prevents admission to the stroke unit or the patient is transferred to high dependency instead (many stroke unit will not accept patients with IV insulin infusions) Perceived increase in staff workload if insulin is administered by IV infusion Belief that there is a lack of research evidence to justify a BGL > 10 as a trigger to treat Sceptism about benefits of administrating insulin for patients with a BGL > 10, e.g. risk of hypoglycemia^b^
	Swallow management	Belief there is lack of robust evidence for effectiveness of non-oral medications such as aspirin
Reinforcement (*n* = 1) Increasing the probability of a response by arranging a dependent relationship, or contingency, between the response and a given stimulus *No, you cannot nurse-initiate PR paracetamol.* [Temperature management] *At the moment we don't have direct access to IV paracetamol in ED, we have to call pharmacy to put an order in.* [Temperature management]	Temperature management	Nurses are unable to administer non-oral paracetamol without a written order^b^
Intentions A conscious decision to perform a behaviour or a resolve to act in a certain way *Example quote not applicable*	*No barriers identified for this behaviour/domain*	*No barriers identified that corresponded with this domain*
Goals (*n* = 3) Mental representations of outcomes or end states that an individual wants to achieve *[Name] has described how busy the ED is and it does add a layer of complexity to the patient when they are on an insulin infusion. [Blood glucose management]*	Triage	Competing priorities in a busy ED environment
	Blood glucose management	Competing priorities in a busy ED environment^b^ Lack of understanding regarding the importance of administering insulin for all stroke patients regardless of diabetic status
Memory, Attention and Decision Processes (*n* = 5) The ability to retain information, focus selectively on aspects of the environment and choose between two or more alternatives *The ED nurses are really good at that [taking temperature on admission], so everyone will get one on admission. It’s just how you remind people at that four hour mark to do it.* [Temperature management] *It's a matter of remembering to request [the formal glucose].* [Blood glucose management]	Triage	Lack of adherence to certain care principles or pathways for stroke patients^b^
	Thrombolysis	Staff overlook documentation of reasons for not administrating rt-PA
	Temperature management	Lack of adherence to certain care principles or pathways for stroke patients^b^
	Blood glucose management	Staff overlook requesting a formal BGL
	Transfer	Competing priorities in a busy ED environment^b^
Environmental Context and Resources (*n* = 30) Any circumstance of a person’s situation or environment that discourages or encourages the development of skills and abilities, independence, social competence, and adaptive behaviour *I think [Name] was worried about increased workload for his department.* [Blood glucose management] *That would be difficult for an infusion to run from coming to ED to ward. We have one working pump at the moment. We have another one that we use for thrombolysis on the ward. So if you're having people coming up on insulin infusions we won't have the equipment.* [Blood glucose management]	Triage	Delays in identifying symptoms of stroke Competing priorities in a busy ED environment^b^ Patient’s mode of presentation at hospital influences triage categories Inconsistent care processes between in-hours and out-of-hours
	Thrombolysis	Delays associated with securing a CT scan^b^ No systems in place to manage stroke calls out-of-hours Delays in authorisation of new protocols/forms by hospital management committees^b^
	Temperature management	Lack of thermometers in ED Lack of knowledge about alternative modes of delivering paracetamol when patient NBM^b^ Hospital regulations set for drug prescribing No hospital protocol for temperature management in stroke patients
	Blood glucose management	Formal BGL testing not routine in current practice No hospital protocol for BGL in stroke patients Hospital initiatives prevent implementation of this care element i.e. cost saving relating to testing of bloods Limited access to BGL machines Lack of insulin infusion pumps Competing priorities in a busy ED environment^b^ Perceived increase in workload for staff administrating insulin to patients by IV infusion Limited time due to competing priorities in a busy environment No hospital protocol for use of insulin infusions in stroke patients Inconsistent use or variation in protocols between ED and stroke unit^b^
	Swallow management	Competing priorities in a busy ED environment^b^ Difficulties with training appropriate staff due to staffing issues, out-of-hours and organisational issues Ineffective systems of communication during staff hand-over on patient transfer from ED to the stroke unit such as lack of documentation of aspirin administration and whether swallow screen done, particularly when the patient failed the screen No seven-day week service provided by speech pathologists
	Transfer	Hospital protocols preclude the transfer of patient undergoing thrombolysis to the stroke unit Ineffective communication between ward staff and bed managers Availability of beds in stroke unit prevent patients from being transferred from ED Staff shortages impacting on bed capacity of the stroke unit Type of stroke may influence patient’s pathway to the stroke unit
Social Influences (*n* = 8) Those interpersonal processes that can cause individuals to change their thoughts, feelings, or behaviours *When there's a protocol and it's the same protocol it's quite easy but when it's different, which it often is … I think there's no continuity …. it falls through the cracks.* [Swallow management]	Thrombolysis	Uncertainty about use of criteria to select patients for rt-PA^b^
	Temperature management	Attitude that changing practices about temperature management requires time
	Blood glucose management	Formal BGL testing is not routine in current practice Clinical opinion overrules guidelines or protocols Negative perception of the value and meaning of other staff roles
	Swallow management	Inconsistent use or variation in protocols between ED and stroke unit^b^ Ineffective systems of communication during patient transfer from ED to stroke unit Nurses lack of confidence to disagree with a doctor’s decision to override a patient’s NBM status^b^
Emotion (*n* = 4) A complex reaction pattern, involving experiential, behavioural, and physiological elements, by which the individual attempts to deal with a personally significant matter or event *We don't want the situation where if there's no beds [in stroke unit], the patient's stuck in ED because they have an insulin infusion.* [Blood glucose management] *I'm slightly concerned they may actually induce hypoglycaemia in the people [for whom] we're trying to adjust the insulin. It's very complicated. I can foresee that the risk for error is quite high.* [Blood glucose management]	Thrombolysis	Uncertainty about use of criteria to select patients for rt-PA^b^
	Blood glucose management	Belief that introducing insulin infusions will have unintended consequences i.e. prevents the admission to the stroke unit or the patient is transferred to a high dependency ward instead Clinical opinion overrules guidelines or protocols
	Swallow management	Nurses lack confidence to disagree with a doctor’s decision to override a patient’s NBM status^b^
Behavioural Regulation (*n* = 3) Anything aimed at managing or changing objectively observed or measured actions *I think a lot of education needs to be provided around that [administering paracetamol at 37.5 °C] because nursing staff always think 38 °C, nothing [no paracetamol] until 38 °C.* [Temperature management] *So I think this will be the most challenging because giving insulin at 10 is not something we would do. That's way outside our practice for normal…* [Blood glucose management]	Temperature management	Nurses routinely and ‘automatically’ treat at a different temperature threshold Staff perception that this action already routine practice
	Blood glucose management	Nurses routinely and ‘automatically’ treat at a different threshold for BGL

*BGL* blood glucose level, *CT* computed tomography, *ED* emergency department, *IV* intravenous, *NBM* Nil by mouth, *rt-PA* recombinant tissue plasminogen activator

^a^‘n’ refers to the number of barriers identified for each domain

^b^Indicates a barrier that was reported for more than one T^3^ trial behaviour

### Step 3: Which intervention components (behaviour change techniques and mode(s) of delivery) could overcome the modifiable barriers and enhance the enablers?

The panel selected appropriate BCTs for the 11 barriers identified for the target behaviour provided (Table 3). There was no TDF domain where the assigned BCTs based on the Cane et al. matrix [20] were viewed as inappropriate by the researchers. Overall, 22 of the selected BCTs for all 11 barriers were based on the Cane et al. matrix [20], with the remaining five selected BCTs based on the Michie et al. matrix [5].

**Table 3:** Behaviour change techniques mapped to the Theoretical Domain Framework identified for intravenous insulin infusion barriers

Domain	Corresponding techniques^a^	Definition of technique
Knowledge	Health consequences	Provide information (e.g. written, verbal, visual) about health consequences of performing the behaviour
	Feedback on behaviour	Monitor and provide informative or evaluative feedback on performance of the behaviour (e.g. form, frequency, duration, intensity)
	Behavioural rehearsal/practice	Prompt practice or rehearsal of the performance of the behaviour one or more times in a context or at a time when the performance may not be necessary, in order to increase habit and skill
	Goal/target specified: behaviour or outcome	Set a goal defined in terms of the behaviour to be achieved
	Self-monitoring	Establish method for the person to monitor and record their behaviour(s) as part of behaviour change strategy
Social/professional role and identity	Social support or encouragement	Advise on, arrange or provide social support (e.g. from friends, relatives, colleagues, ‘buddies’ or staff) or non-contingent praise or reward for performance of the behaviour. It includes encouragement and counselling, but only when it is directed at the behaviour
	Salience of consequences	Use methods specifically designed to emphasise the consequences of performing the behaviour with the aim of making them more memorable (goes beyond informing about consequences)
	Anticipated regret	Induce or raise awareness of expectations of future regret about performance of the unwanted behaviour
	Social and environmental consequences	Provide information (e.g. written, verbal, visual) about social and environmental consequences of performing the behaviour
	Comparative imagining of future outcome	Prompt or advise the imagining and comparing of future outcomes of changed versus unchanged behaviour
	Pros and cons	Advise person to identify and compare reasons for wanting (pros) and not wanting (cons) to change behaviour
	Persuasive communication	Credible source presents arguments in favour of the behaviour
	Feedback on behaviour	Monitor and provide informative or evaluative feedback on performance of the behaviour (e.g. form, frequency, duration, intensity)
	Goal setting (behaviour)	Set a goal defined in terms of the behaviour to be achieved
	Action planning (including implementation intentions)	Prompt detailed planning of performance of behaviour (must include ≥ one of context, frequency, duration and intensity). Context may be environmental (physical or social) or internal (physical, emotional or cognitive)
Memory, Attention and Decision Processes	Planning, implementation	Prompt detailed planning of the behaviour goal (including at least one of context, frequency, intensity and duration of performance)
	Prompts, triggers, cues	Use environmental, social or internal stimuli to prompt or cue performance of wanted behaviour or non-performance of unwanted behaviour
Environmental context and resources	Restructuring the social environment	Change, or advise to change the social environment in order to facilitate performance of the wanted behaviour or create barriers to the unwanted behaviour (other than prompts/cues, rewards and punishments)
	Prompts/cues	Introduce or define environmental or social stimulus with the purpose of prompting or cueing the behaviour. The prompt or cue would normally occur at the time or place of performance
	Avoidance/changing exposure to cues for the behaviour	Advise on how to avoid exposure to specific social and contextual/physical cues for the behaviour, including changing daily or weekly routines
	Environmental changes (e.g. objects to facilitate behaviour)	Change the environment in order to facilitate the target behaviour (other than prompts, rewards and punishments, e.g. choice of food provided)
Social Influences	Social comparison	Explicitly draw attention to others’ performance to elicit comparisons
	Social support or encouragement (general)	Advise on, facilitate or provide development of general social support for the behaviour (e.g. friends, relatives, colleagues, ‘buddies’ or staff)
	Information about others approval	Provide information about what other people think about the behaviour. Clarifies whether others will like, approve or disapprove of what the person is doing or will do
	Social support (emotional)	Advise on or facilitate development of emotional social support for performing the behaviour
	Social support (practical)	Advise on or facilitate development of practical help for achieving the behaviour
	Modelling or demonstrating the behaviour	Provide an example for people to aspire to or imitate
Emotion	Reduce negative emotions	Advise on ways of reducing negative emotions to facilitate performance of the behaviour
	Coping skills	Analyse problem and generate or select solutions that include overcoming barriers and increasing facilitators
Behavioural Regulation	Self-monitoring of behaviour	Establish method for person to monitor and record their behaviour(s) as part of a behaviour change strategy

^a^Label as per matrix by Cane et al. [20]

#### Generating a final set of BCTs

The findings from the researcher panel were then applied to the remaining 11 behaviours to generate a final set of BCTs to apply to all the behaviours. Two TDF domains (*beliefs about capabilities* and *reinforcement*) were not represented by any of the 11 barriers used in the researcher panel; therefore, selection of the most appropriate BCTs was based on the triallists experience [6]. The final set of BCTs (*n* = 27) are reported by general implementation intervention components in Table 4. Some techniques, e.g. *action planning* and *coping skills,* were classified into more than one relevant implementation intervention component.

**Table 4:** Theory-informed implementation intervention: components by selected behavioural change techniques

Implementation intervention component	Selection of behavioural change techniques
Multidisciplinary barrier and enabler workshops for ED, stroke unit and endocrine clinicians	Goal/target specified: behaviour or outcome
	Social and environmental consequences
	Restructuring the social environment
	Environmental changes (e.g. objects to facilitate behaviour)
	Social support (practical)
	Social support (emotional)
	Planning, implementation
	Action planning
	Goal setting (behaviour)
Interactive and didactic education programme for ED and stroke unit clinicians	Health consequences
	Behavioural rehearsal/practice
	Social and environmental consequences
	Salience of consequences
	Feedback on behaviour
	Focus on past success
	Social comparison
	Reduce negative emotions
	Anticipated regret
	Coping skills
	Comparative imaging of future outcomes
Use of local clinical opinion leaders	Verbal persuasion to boost self-efficacy
	Persuasive communication
	Pros and cons
	Modelling/demonstration of the behaviour
	Anticipated regret
	Social comparison
	Information about others’ approval
Reminders	Prompts/cues
	Avoidance/changing exposure to cues for the behaviour
Site support	Self-monitoring
	Self-reward
	Social support or encouragement
	Coping skills
	Action planning
	Goal setting

Technique may be classified to more than one implementation intervention component, e.g. action planning

## Discussion

The barriers that hospital staff believed to be likely to influence the implementation of the T^3^ Trial clinical protocol were mapped to 13 TDF domains. These domains were used to guide the content of an implementation intervention consisting of 27 BCTs. It is anticipated that by using this systematic, theory-based approach to inform the content of an implementation intervention the potential for effectiveness in changing behaviour will be optimised. Researcher opinion, together with the existing research evidence base, was used to refine the theoretically driven intervention framework by selecting appropriate BCTs and suggesting strategies to operationalise the BCTs in an ED context. One of the few studies that used the TDF and BCTs to define the content of an intervention to change patient’s compliance in bronchiectasis used an adapted scoring system from Michie et al. to select BCTs from a longer list [22]. Experts classified BCTs into one of the following categories which then generated the scores: agreed use, agreed non-use, disagreement and uncertain. The validity of using such criteria in the BCT selection process was not reported by the authors, indicating that further research is required in this area. The use of stakeholder opinion has been used in another study to define how the proposed intervention could be delivered as opposed to selecting the BCTs [22]. This process of incorporating the views of researchers with experience of the clinical context and knowledge of the clinical intervention is likely to enhance the clinical acceptability of the implementation intervention. The BCTs selected by the researchers aligned with the BCTs recommended by the matrix, suggesting that this is a valuable tool to use to highlight relevant BCTs.

### Limitations

The final set of BCTs was generated using the researcher panellists’ selections based on one target behaviour. This assumes that the BCTs considered appropriate for one behaviour have applicability to target similar barriers associated with the other behaviours. Potentially, appropriate BCTs to target different barriers for the other behaviours may not have been included. However, the purpose of the researcher panel was to develop a final set of BCTs that could be used for each of the behaviours. It was considered resource-intensive to apply this selection process for all 12 behaviours in this complex intervention. Acknowledging this limitation, this approach should be considered valid and reliable as a theoretical framework was used to develop the implementation intervention and nearly all the relevant TDF domains (11 out of 13) common to the 12 behaviours were represented by the behaviour used as an example in the researcher panel. The considerable time and resources required in the intervention development process have been raised elsewhere [6] and have implications for applying for research funding, whereby funding bodies often expect the intervention to be fully developed on application, which is usually not possible without separate pre-trial funding, usually difficult to secure.

### Strengths

The barrier and enabler workshops allowed comprehensive identification of relevant barriers and enablers by hospital staff, providing data for each of the T^3^ Trial behaviours. This ensured that all the individual elements of the T^3^ Trial clinical intervention were addressed, a necessity for complex interventions. Using the TDF framework enabled the classification of barriers to BCTs required for each T^3^ Trial behaviour. The TDF is readily being used by researchers to explore behaviour change but few describe the process of how to use theoretical frameworks when developing implementation interventions. Describing the process in steps ensured transparency and replicability of the method that could be used to develop similar implementation interventions for stroke treatments or guideline implementations across different conditions. The use of researchers incorporated well-informed judgment, acknowledged to be an important part of the process, especially for complex interventions [35]. The enabler data provided the researchers with valuable context-specific information to assist in the decision-making process. Reporting the BCT’s using a standard taxonomy for by each T^3^ Trial behaviour has produced a well-described, reproducible and testable implementation intervention.

### Further research

This study used a pragmatic approach to selecting BCTs that involved the T^3^ investigators who were familiar with the clinical protocols and clinical context. However, there is little evidence on who is best placed to make these judgments and what methods should be used. Michie et al. has developed the ‘APEASE’ criteria (A: Affordability; P: Practicability; E: Effectiveness/cost-effectiveness; A: Acceptability; S: Side-effects/Safety; E: Equality). However, it is unlikely that this information will be available for all the BCTs [7]. The T^3^ Trial is one of the few studies that have reported BCTs that were considered inappropriate for use (Table 3). Knowing more about the BCTs that are inappropriate for certain clinical contexts or even healthcare settings generally would be useful to make the overall pool of BCTs to choose from more relevant and straightforward. The Cane et al. matrix [20] is limited in suggesting BCTs for two of the TDF domains (social/professional role and identity; memory, attention and decision process). Therefore, further work should be conducted to assign more BCTs to these two domains. We acknowledge that research is currently underway which aims to link BCTs with theoretical mechanisms to better understand how interventions exert their effect and how to apply theory in implementation intervention development [36]. It is anticipated the approach used in this paper to develop and report an implementation intervention may contribute to the advancement of use of theory to guide intervention development. The TDF was a useful tool to map barriers to theoretical domains; however, as highlighted in this paper, inconsistencies between researchers can arise. Therefore, further work to clarify TDF domain definitions and to provide example barriers for each domain would complement this mapping process and minimise disagreements.

## Conclusion

The TDF was successfully applied in all steps of developing an implementation intervention for the T^3^ Trial clinical protocols. The use of researcher opinion was valuable for the BCT selection process in terms of incorporating research evidence and well-informed judgment and incorporating the important practical issues of feasibility and acceptability. However, further recommendations are needed to advance understanding of who is best placed to inform implementation intervention development, and how best to incorporate this well-informed judgment. There is also a need to devise criteria for use in this BCT selection process. It is recommended that BCTs are classified by recognised implementation intervention components to facilitate generalisability and sharing across different conditions and settings.

## Additional files

Additional file 1: Resource 1: barrier extracts and TDF definitions. (DOCX 23 kb)

Additional file 2: Resource 2: domains with corresponding BCT and definitions. (DOCX 25 kb)

Additional file 3: Resource 3: technique definition and examples. (DOCX 71 kb)

Additional file 4: Resource 4: enabler data from the barrier and enabler workshops. (DOCX 26 kb)

Additional file 5: Resource 5: Cochrane Effective Practice and Organisation of Care [EPOC] reviews. (DOCX 25 kb)
