# Supplementary material for: Development of a theory-informed implementation intervention to improve the triage, treatment and transfer of stroke patients in emergency departments using the Theoretical Domains Framework (TDF): the T3 Trial
Source: Implement Sci. 2017 Jul 17;12:88. doi: 10.1186/s13012-017-0616-6 (PMC5513365; doi:10.1186/s13012-017-0616-6)
Supplement: Supplementary file 1 — Resource 1: barrier extracts and TDF definitions. (DOCX 23 kb) [file 13012_2017_616_MOESM1_ESM.docx]

Table 1 Barrier descriptions and quote with corresponding Theoretical Domain Framework (TDF) Domain

| **Barrier** | **Description** *(Example quote)* | **TDF Domain (See Table 2 for definitions)** |
| --- | --- | --- |
| **Negative attitudes towards the use of insulin infusions** | Negative attitudes towards the use of insulin infusions and introducing new insulin algorithms. Includes unintended consequences; the IV infusion resulting in the patient actually staying in ED and preventing immediate transfer to the stroke unit or having to go to HDU.  *“it's going to be a major issue, major issue because of the fact*  *that most wards will say we don't like them, we can't do them, we won't do the”* | Beliefs about consequences  Emotion  Optimism |
| **Staff perceive increase workload associated with patients on an insulin infusion** | Perceived increase workload associated with patients on an insulin infusion.  *“I think [Name] was worried about increased workload for his*  *department.”* | Environmental context and resources  Beliefs about consequences |
| **Staff faced with competing priorities in a busy environment** | Staff time pressured.  *“[Name] has described how busy the ED is and it does add a*  *layer of Complexity to the patient when they are on an insulin*  *dextrose infusion”* | Environmental context and resources  Goals |
| **Nurses do not understand the importance of administering insulin for all stroke patients regardless of diabetic status** | The need to consider other patient characteristics i.e. whether they are diabetic.  *Facilitator: “What's your normal threshold?*  *Staff member: “It depends if they're diabetic or non diabetic. “* | Knowledge  Memory, attention and  decision processes |
| **Lack of infusion pumps** | Lack of infusion pumps.  *“We have one working pump at the moment. We have another*  *one that we use for thrombolysis on the ward because it's been*  *fast tracked. So if you're having people coming up on*  *insulin infusions we won't have the equipment.”* | Environmental context and resources |
| **Staff believe there is a lack of research evidence to justify a BGL > 10 as a trigger to treat** | Lack of research evidence around the outcomes of monitoring and treatment of BSL in stroke.  *“But with the unimpressive previous studies with stroke I don't*  *think any of the consultants here feels that it's particularly*  *worth pushing”* | Beliefs about consequences |
| **Lack of knowledge and/or skill about the process of administering insulin infusion.** | Lack of knowledge and/or skill about the process of administering insulin infusion.  *“It’s the wards, there’s a lot of wards not used to running*  *infusions”*  *“We have a high staff turnover, so they could all be educated in*  *doing it. It's a pity we don't have a Clinical Nurse*  *Educator five days a week to support that, but anyway.”* | Knowledge  Skills |
| **No hospital protocol and/or support for the use of insulin infusions in stroke patients** | This includes references to the lack of consistency. Hard to be consistency, no guidance with regards to the dose. Includes lack of organisational support.  *“that there isn't a protocol [……]I think when people are on*  *top of it it's fine and when there's a protocol and it's the same*  *protocol it's quite easy but when it's different, which it often it is*  *for different doctor charts, for example. I think there's no*  *continuity, there's no streamline, then it falls through the*  *cracks.* | Environmental context and resources |
| **Nurses routinely treat at a different threshold for BGL** | Do not normally treat at >10. Differences between existing and trial i.e. don’t start insulin infusion without glucose infusion.  “*So I think this will be the most challenging because giving insulin at 10 is not something we would do. That's*  *way outside our practice for normal…”* | Behavioural Regulation |
| **Negative perception of the value and meaning of other staff roles** | That some are not regarded as part of the team. People are less busy that others.  *“It gives the endocrinology registrar something to do”* | Social influences |
| **Skeptism towards the intervention with regards to risks and benefits** | There is uncertainty of the benefits of treating glucose at this level. Risk of hypoglycaemia, number of IV accesses, response to treatment. Benefit/risk ratio. Includes relatives and patient.  *“I think you're right there is a fear of hypoglycaemia, especially*  *in stroke patients who are obviously a slightly different group*  *who may be meal by mouth, not be getting any feeding at all.*  *So a BSL of 10.1 and then putting them on insulin infusion when*  *they're not eating anything I think starts to become*  *also a little bit of a concern that way.”* | Beliefs about consequences  Social influences  Knowledge |
| **Variation in practice** | The need for the same protocol across ED and the SU. Different unit having the same protocols.  *“There's always the staff that want a different version on what*  *they want to give”*  *“we obviously need to do a little bit of work in the stroke unit so*  *they can receive patients with an insulin infusion.”* | Social influences  Environmental context and resources  Social Professional role and identity |

**Table 2 Theoretical Domain Framework Domain Definitions**

| **Domain** | **Definition** |
| --- | --- |
| Behavioural Regulation | Anything aimed at managing or changing objectively observed  or measured actions |
| Beliefs about Consequences | Acceptance of the truth, reality, or validity about outcomes of a  behaviour in a given situation |
| Beliefs about Capabilities | Acceptance of the truth, reality, or validity about an ability, talent, or facility that a person can put to constructive use |
| Emotion | A complex reaction pattern, involving experiential, behavioural,  and physiological elements, by which the individual attempts to deal with a personally significant matter or event |
| Environmental Context and Resources | Any circumstance of a person's situation or environment that  discourages or encourages the development of skills and  abilities, independence, social competence, and adaptive behaviour |
| Goals | Mental representations of outcomes or end states that an  individual wants to achieve |
| Intentions | A conscious decision to perform a behaviour or a resolve to act in  a certain way |
| Knowledge | An awareness of the existence of something |
| Memory, Attention and Decision Processes | The ability to retain information, focus selectively on aspects  of the environment and choose between two or more alternatives |
| Optimism | The confidence that things will happen for the best or that desired goals will be attained |
| Reinforcement | Increasing the probability of a response by arranging a dependent relationship, or contingency, between the response and a given stimulus |
| Social influences | Those interpersonal processes that can cause individuals to  change their thoughts, feelings, or behaviours |
| Social/Professional Role and Identity | A coherent set of behaviours and displayed personal qualities of an individual in a social or work setting |
| Skills | An ability or proficiency acquired through practice |
|  |  |
