# Supplementary material for: Development of a theory-informed implementation intervention to improve the triage, treatment and transfer of stroke patients in emergency departments using the Theoretical Domains Framework (TDF): the T3 Trial
Source: Implement Sci. 2017 Jul 17;12:88. doi: 10.1186/s13012-017-0616-6 (PMC5513365; doi:10.1186/s13012-017-0616-6)
Supplement: Supplementary file 2 — Resource 2: domains with corresponding BCT and definitions. (DOCX 25 kb) [file 13012_2017_616_MOESM2_ESM.docx]

**Table 3 TDF Domains with corresponding techniques and definitions**

| **Domain** | **Corresponding techniques** | **Definition of technique** |
| --- | --- | --- |
| **Behavioural Regulation** | Self-monitoring of behaviour | Establish a method for the person to monitor and record their behavior(s) as part of a behavior change strategy |
| **Beliefs about consequences** | Emotional consequences | Provide information (e.g. written, verbal, visual) about emotional consequences of performing the behavior |
|  | Salience of consequences | Use methods specifically designed to emphasise the consequences of performing the behaviour with the aim of making them more memorable (goes beyond informing about consequences) |
|  | Covert sensitization | Advise to imagine performing the unwanted behavior in a real-life situation followed by imagining an unpleasant consequence |
|  | Anticipated regret | Induce or raise awareness of expectations of future regret about performance of the unwanted behavior |
|  | Social and environmental consequences | Provide information (e.g. written, verbal, visual) about social and environmental consequences of performing the behavior |
|  | Comparative imaging of future outcome | Prompt or advise the imagining and comparing of future outcomes of changed versus unchanged behaviour |
|  | Vicarious reinforcement | Prompt observation of the consequences (including rewards and punishments) for others when they perform the behavior |
|  | Threat | Inform that future punishment or removal of reward will be a consequence of performance of an unwanted behavior (may include fear arousal) |

|  | Pros and Cons | Advise the person to identify and compare reasons for wanting (pros) and not wanting to (cons) change the behavior |
| --- | --- | --- |
|  | Covert conditioning | Advise to imagine performing the wanted behavior in a real-life situation followed by imagining a pleasant consequence |
|  | Self-monitoring of behaviour | Establish a method for the person to monitor and record their behavior(s) as part of a behavior change strategy |
|  | Persuasive communication | Credible source presents arguments in favour of the behaviour |
|  | Feedback on behavior | Monitor and provide informative or evaluative feedback on performance of the behavior *(e.g. form, frequency, duration, intensity)* |
| **Emotion** | Reduce negative emotions | Advise on ways of reducing negative emotions to facilitate performance of the behavior |
|  | Emotional consequences | Provide information (e.g. written, verbal, visual) about emotional consequences of performing the behavior |
|  | Self-assessment of affective consequences | After attempts at performing the behaviour, prompt assessment of feelings about performing the behaviour |
|  | Social support (emotional) | Advise or agree on how to perform the behavior |
|  | Stress management | This may involve a variety of specific techniques (e.g., progressive relaxation) which do not target the behaviour directly but seek to reduce anxiety and stress to facilitate the performance of the behaviour |
|  | Coping skills | Analyse the problem and generate or select solutions that include overcoming barriers and increasing facilitators |
| **Environmental context and resources** | Restructuring the social environment | Change, or advise to change the social environment in order to facilitate performance of the wanted behavior or create barriers to the unwanted behavior (other than prompts/cues, rewards and punishments) |
|  | Discrimitive (learned) cue | Identify an environmental stimulus that has been repeatedly associated with contingent reward for specified behaviour |
|  | Prompts/cues | Introduce or define environmental or social stimulus with the purpose of prompting or cueing the behavior. The prompt or cue would normally occur at the time or place of performance |
|  | Avoidance/changing exposure to cues for the behaviour | Advise on how to avoid exposure to specific social and contextual/physical cues for the behavior, including changing daily or weekly routines |
|  | Environmental changes (e.g. objects to facilitate behaviour) | Change the environment in order to facilitate the target behavior (other than prompts, rewards and punishments, e.g. choice of food provided) |
| **Goals** | Goal setting (outcome) | Set or agree on a goal defined in terms of a positive outcome of wanted behavior |
|  | Goal setting (behaviour) | See Goal setting (outcome) |
|  | Review of outcome goal(s) | See Goal setting (outcome) |
|  | Review behaviour goals | Review behavior goal(s) jointly with the person and consider modifying goal(s) or behavior change strategy in light of achievement. This may lead to re-setting the same goal, a small change in that goal or setting a new goal instead of (or in addition to) the first, or no change |
|  | Action planning (including implementation intentions) | Prompt detailed planning of performance of the behavior (must include at least one of context, frequency, duration and intensity). Context may be environmental (physical or social) or internal (physical, emotional or cognitive) |
| **Knowledge** | Health consequences | Provide information (e.g. written, verbal, visual) about health consequences of performing the behavior |
|  | Biofeedback | Provide feedback about the body *(e.g. physiological or biochemical state)* using an external monitoring device as part of a behavior change strategy |
|  | Antecedents | Provide information about antecedents  (*e.g. social and environmental situations and events, emotions, cognitions)* that reliably predict performance of the behaviour |
|  | Feedback on behaviour | Monitor and provide informative or evaluative feedback on performance of the behavior *(e.g. form, frequency, duration, intensity)* |
| **Memory, Attention and Decision Processes** | Planning, implementation | Prompt detailed planning of the behaviour goal (including at least one of context, frequency, intensity and duration of performance) |
|  | Prompts, triggers, cues | Use environmental, social or internal stimuli to prompt or cue performance of wanted behaviour or non-performance of unwanted behaviour |
|  | Self-monitoring | Instruct self-recording of specified behaviour/s (with or without associated thoughts, emotions, situations) as part of a behaviour change strategy |
| **Optimism** | Verbal persuasion to boost self-efficacy | Tell the person that they can successfully perform the wanted behavior, arguing against self-doubts and asserting that they can and will succeed |
| **Social influences** | Social comparison | Draw attention to others’ performance to allow comparison with the person’s own performance *Note:* *being in a group setting does not necessarily mean that social comparison is actually taking place* |
|  | Social support or encouragement | Advise on, arrange or provide social support *(e.g. from friends, relatives, colleagues,’ buddies’ or staff)* or non-contingent praise or reward for performance of the behavior*.* It includes encouragement and counselling, but only when it is directed at the behavior |
|  | Information about others’ approval | Provide information about what other people think about the behavior. The information clarifies whether others will like, approve or disapprove of what the person is doing or will do |
|  | Social support (emotional) | Advise on, arrange, or provide emotional social support *(e.g. from friends, relatives, colleagues, ‘buddies’ or staff)* for performance of the behavior |
|  | Social support (practical) | Advise on, arrange, or provide practical help *(e.g. from friends, relatives, colleagues, ‘buddies’ or staff)* for performance of the behavior |
|  | Vicarious reinforcement | Facilitate observation of the consequences for others when they perform the target behaviour |
|  | Restructuring of social environment | Change, or advise to change the social environment in order to facilitate performance of the wanted behavior or create barriers to the unwanted behavior (other than prompts/cues, rewards and punishments) |
|  | Modelling/demonstration of behaviour | Provide an observable sample of the performance of the behaviour, directly in person or indirectly e.g. via film, pictures, for the person to aspire to or imitate |
| **Social/professional role and identity** | Social support or encouragement | Advise on, arrange or provide social support *(e.g. from friends, relatives, colleagues,’ buddies’ or staff)* or non-contingent praise or reward for performance of the behavior*.* It includes encouragement and counselling, but only when it is directed at the behavior |
| **Skills** | Graded tasks | Set easy-to-perform tasks, making them increasingly difficult, but achievable, until behavior is performed |
|  | Behavioural rehearsal/practice | Prompt practice or rehearsal of the performance of the behavior one or more times in a context or at a time when the performance may not be necessary, in order to increase habit and skill |
|  | Habit reversal | Prompt rehearsal and repetition of an alternative behavior to replace an unwanted habitual behavior |
|  | Body changes | Alter body structure, functioning or support directly to facilitate behavior change |
|  | Habit formation | Prompt rehearsal and repetition of the behavior in the same context repeatedly so that the context elicits the behavior |
|  | Goal/target specified: behaviour or outcome | Set a goal defined in terms of the behaviour to be achieved |
|  | Self-monitoring | Establish a method for the person to monitor and record their behavior(s) as part of a behavior change strategy |
|  | Rewards; incentives (self-evaluation) | Inform that performance will be rewarded contingent on behaviour in the future |
| Behavior change techniques listed were informed by 1) previous work that link BCTs from the taxonomy to the refined TDF (Cane et al 2015) 2) a matrix that mapped behaviour change techniques to the theoretical domains, based on expert consensus about effectiveness for behaviour change (Mitchie, 2008)  *Red indicates that the matrix used to map BCT to the TDF domain was based on Mitchie et al 2008 | | |
