# Supplementary material for: Development of a theory-informed implementation intervention to improve the triage, treatment and transfer of stroke patients in emergency departments using the Theoretical Domains Framework (TDF): the T3 Trial
Source: Implement Sci. 2017 Jul 17;12:88. doi: 10.1186/s13012-017-0616-6 (PMC5513365; doi:10.1186/s13012-017-0616-6)
Supplement: Supplementary file 4 — Resource 4: enabler data from the barrier and enabler workshops. (DOCX 26 kb) [file 13012_2017_616_MOESM4_ESM.docx]

Raw Qualitative Data

| **Hospital** | **Qualitative extracts relating to facilitators and practical issues**  The care element is: Administer insulin to all patients with BGL > 10 mMol/L within one hour |
| --- | --- |
| Hospital 1 | ”Right, okay, all right, well we can certainly work out a protocol. We've got  standing protocols already, so looking at those to see whether they're suitable.”  “But I think basically probably the best protocol system is that you have a protocol that you  start and if it's working, you keep running it. If you don't like what's happening, you ring up  and say, can you come and help us.”  Facilitator: “And have a bit of a chat perhaps with our endocrinologist [T3].”  Participant: “Yeah, sure.” |
| Hospital 2 | Participant 1 : “So we need to work out whether that's - is there a possibility of getting any  More equipment. How expensive are these pumps?”  Participant 2: “A couple of grand and we don't have the funds at a ward level to be funding  that. I can't get one for [unclear] or the endocrinology patients at the moment.”  “The endocrinologists here, I think they realise they were missing a whole lot of  diabetics so getting involved early.”  Female: “Have other sites highlighted this concern?”  Facilitator: “Some sites, yes.”  “We should be using our clinical judgement.”  “So it's unfortunate that no-one from endocrinology is here at the moment but I think - I don't know that they'll be - certainly when we've discussed this with them in the past based on the results of the QASC trial then they were not excited about those sorts of - that sort of control because well it meant looking at what sort of intervention were we going to have and intravenous insulin infusions are…not risk free”  “Well they're thousands and thousands of dollars and the organisation has just introduced a  whole new set as well but things like that where are they coming from? “  “I guess we can try and re-open discussions with our endocrine.”  “They weren't [endocrine] difficult, they just didn't agree with the intervention that's been  proposed with this trial and neither did we to be perfectly honest at the time. Having said  that we're all aware of the good outcomes in I mean I think we should re-open some  discussions locally with our endocrine team and see if we can get a protocol going that's  sort of sensible but I think it's probably not going to involve insulin infusions in the vast  majority of cases.” |
| Hospital 3 | ”I don't see why we don't have insulin infusions on the ward. This is my personal  view, everywhere I've worked it's not been an issue anywhere else, in this  hospital, it is an issue around insulin infusions on the ward.”  “I've asked Gail who's our CNC for diabetes to have a look at it, because really I  don't understand why it's such an issue.”  “I don't think it's a problem we can't overcome, I just need to get [name of  endocrinologist’s] view on it We did communicate with him ages ago but I think it was  probably when we were first discussing the monitoring stage. I think the intravenous thing  was a big issue, [name of endocrinologist’s] despises the sliding scale insulin and I think  endocrinologists generally have gone off sliding scales which I think is fair enough.”  “We would like to see a protocol that someone else is using…”  “Or perhaps your endocrinologist could to talk to our endocrinologist.”  “We have a high staff turnover, so they could all be educated in doing it. It's a pity we don't  have a [CNE] five days a week to support that, but anyway.”  “And you'd need a clear pathway for the nursing staff to follow nurses could be educated on  a stroke unit to take an infusion from ED”  “It needs ... registered nurses.”  “So I'm presuming that will be a big part of the education, because it's going to be a new…” |
| Hospital 4 | “Well, I think very aggressive therapy has been universally found now to be  harmful in most situations, with one or two exceptions in surgical and ICUs. But  certainly most of ischemic events [both had a function in stroke], very  aggressive control is probably - and that's [unclear] less than 6 is thought to be  no-go, but we know the national level is 10. We believe that that's probably a  little high. [So naturally within the community were aiming] for 8, but I know  there's a lot of evidence about exactly what the precise level [unclear] is. I think  there's good evidence that high sugars, certainly over 15, are harmful. [Unclear]  I think aiming for sugars [between 4 and 8], and [avoiding] hypoglycaemia is  really the aim.”  “I think there probably with this would need to be some education medically” |
| Hospital 5 | “I think we'd have to have a very clear protocol written for us so that everybody's doing the  same thing all the time”  “Which reminds me, I've been meaning to talk to you. We're running a group to reorganize  glucose, insulin charts et cetera, the insulin protocols. We were wanting someone from ED  involved in it. We can work on - work off this as well and the same time.”  “In stroke, is there evidence that control - keeping sugars within their range - clearly the  sicker stroke is going to have a higher sugar aspire systemic inflammatory response. Have  we shown that aggressively keeping sugar in normal ranges is of benefit to patients?  It doesn't hurt us to try to be uniform in our approach.”  “I'm not saying we don't care. It's just I'm saying that we don't have a specific threshold to  say everybody will do something at this level. We're all just slightly different and nobody's  looked at trying to make sure we all conform” |
| Hospital 8 | Facilitator: “So yeah, hyperglycaemia is a risk but so is hypoglycaemia. But the experience in  the QASC trial was that that was actually safe and they did well and they work out  algorithms that worked extremely well in practice. I suppose it's sort of an education and  enthusiasm point of view. I suspect it will make quite a difference. Do you think it would  help if we sort of just spent a bit of time reiterating that and going through it and going over  the evidence from QASC about how many hypos were had and things like that?”  Participant: “Well, that would help but just also if we had some actual - how much to give  and when…”  “What are other people doing?”  Facilitator: “It depends on the site. Some sites are going with if it goes above 10, they're  Going to start an insulin infusion”  “I mean if we go back, there were no issues when we ran it in the stroke unit with QASC  trials. Okay, because that may well have not been a lot of patients but we'll need to look at  our previous how we went with that” |
| Hospital 9 | “That was what happened during the QASC when we did it. We used insulin infusions and got endocrine involved but then that wasn't used throughout the hospital. So we defaulted back to the old method, but we need to revisit it.”  “From my experience I would say that I wouldn't try to educate the doctors too  much….I mean I think that…If we're going to drive it then if they listen to us…”  “I think the nursing staff should be educated because the turnover is much less and in my  experience they're much more keen to take it up” |
| Hospital 11 | “It's going to take a lot of work. We'll need quite a lot of education around it.”  “We'd need the equipment and a lot of education.” |
| Hospital 12 | “It would be nice if we had an idea on how many numbers this would be over a year at our  Facility. But if it's tiny numbers, because it’s going to be a distinct change in practice, so if  it’s tiny numbers, I guess the nursing staff will have to figure out whether they can cope. But  if it’s anything more than tiny, it’s probably a big change in ward practice.”  “Well, I would do it as part of the doctor education, which will probably go out one ear but  then they'll remember when the nurse comes and bothers them. we’d have get some  pumps or have a pump on the ward mostly all the time. We only have one pump.” |
| Hospital 13 | “so we'll give you a bit of extra in-servicing training if necessary”  “We have got like a 150 nursing staff so if we're talking about training them, we have to  have a good approach to make sure that they're on every shift, for every patient that you  need it for to make it realistic.” |
