# Supplementary material for: Development of a theory-informed implementation intervention to improve the triage, treatment and transfer of stroke patients in emergency departments using the Theoretical Domains Framework (TDF): the T3 Trial
Source: Implement Sci. 2017 Jul 17;12:88. doi: 10.1186/s13012-017-0616-6 (PMC5513365; doi:10.1186/s13012-017-0616-6)
Supplement: Supplementary file 5 — Resource 5: Cochrane Effective Practice and Organisation of Care [EPOC] reviews. (DOCX 25 kb) [file 13012_2017_616_MOESM5_ESM.docx]

**Cochrane EPOC reviews evidence to inform intervention components (adapted from Tavender et al 2015)**

| **Cochrane review topic** | **Definition** | **Mechanism of action and practicality** | **Key findings** | **Effect sizes** | **Proposed implications for intervention components** |
| --- | --- | --- | --- | --- | --- |
| Continuing education meetings and workshops ^1^ | Participation of healthcare providers in conferences, lectures, workshops or traineeships | Didactic meetings target knowledge at the individual healthcare professional/peer group level. Interactive workshops target knowledge, attitudes and skills. Practicalities: commonly used with the main cost related to the release time for healthcare professionals and feasible in most settings. | Educational meetings alone or combined with other interventions can improve professional practice and the patient healthcare outcomes. The effect on professional practice tended to be small and varied between studies, and the effect on patient outcomes was generally less. It is not possible to explain the observed differences in effect with confidence but it appeared that higher attendance at the meetings was associated with greater effects, that mixed interactive and didactic education was more effective than either alone, and that the effects were less for more complex behaviours and less serious outcomes. | 81 randomised controlled trials (11,000+ health professionals). Median absolute improvement in care of 6.0 % (IQR +1.8 % to +15.3 %). | Mixed interactive workshops and didactic education. [Note: may have smaller effects as mTBI is seen as a ‘less serious’ condition]. |
| Local opinion leaders ^2^ | Use of providers nominated by their colleagues as ‘educationally influential’ | Target: knowledge, attitudes and social norms of their peer group. Dependent on the existence of intact social networks within professional communities. Practicalities: resources required include cost of the identification method, training of opinion leaders and additional service costs | Opinion leaders alone or in combination with other interventions may successfully promote evidence-based practice, but effectiveness varies both within and between studies. These results are based on heterogeneous studies differing in terms of type of intervention, setting, and outcomes measured. In most of the studies, the role of the opinion leader was not clearly described, and it is therefore not possible to say what the best way is to optimise the effectiveness of opinion leaders | 18 randomised controlled trials (296 hospitals and 318 primary care physicians). Median absolute improvement in care of 12 % (IQR +6.0 % to 14.5 %). | Local opinion leaders (clinical champions) to be nominated at each site and their characteristics and role to be clearly described. |
| Printed educational materials^3^ | Distribution of published or printed recommendations for clinical care including clinical practice guidelines, audio-visual materials and electronic publications. The materials may have been delivered personally or through mass mailings. | Target: knowledge and potential skill gaps of individual healthcare professionals. Can be used to target motivation when written as a ‘persuasive communication’ but little evidence of being used in this way. Practicalities: commonly used and relatively low cost and feasible in most settings | Printed educational materials when used alone and compared to no intervention may have a small beneficial effect on professional practice outcomes. There is insufficient information to reliably estimate the effect of PEMs on patient outcomes, and clinical significance of the observed effect sizes is not known. The effectiveness of PEMs compared to other interventions, or of PEMs as part of a multifaceted intervention, is uncertain | 14 randomised controlled trials and 31 interrupted time series studies (ITS). Median absolute risk difference in categorical practice outcomes was 0.02 when PEMs were compared to no intervention (range from 0 to +0.11). | Clinical guideline and key research publications to be provided. |
| Audit and feedback ^4^ | Any summary of clinical performance of healthcare over a specified period of time to change health professional behaviour as indexed by objectively measured professional practice in a healthcare setting or healthcare outcomes. | Target: ‘healthcare provider/peer groups’ perceptions of current performance levels and useful to create cognitive dissonance within healthcare professionals as a stimulus of behaviour change’. Practicalities: resources required to deliver audit and feedback including data extraction, analysis and dissemination costs. Feasibility dependent on availability of meaningful routine administrative data for feedback. | Audit and feedback generally leads to small but potentially important improvements in professional practice. The effectiveness of audit and feedback seems to depend on baseline performance and how the feedback is provided. Audit and feedback may be most effective when: (1) the health professionals are not performing well to start out with, (2) the person responsible for the audit and feedback is a supervisor or colleague, (3) it is provided more than once, (4) it is given both verbally and in writing and (5) it includes clear targets and an action plan. | 140 randomised controlled trials. Median adjusted RD was 4.3 % (IQR 0.5 % to 16 %). | Regular audit and feedback provided by senior work colleague, provided in verbal and written format. Clear targets and action plan provided. [Note: Not feasible as ED rarely has routine administrative data for the behaviours targeted in this intervention.] |
| On-screen point of care computer reminders^5^ | Patient or encounter specific information, provided verbally, on paper or on a computer screen, which is designed or intended to prompt a health professional to recall information. | Target: prompt health professionals to remember to do important things during patient interaction. Practicalities: resources necessary vary across the delivery mechanism. | Point of care computer reminders generally achieve small to modest improvements in provider behaviour. A minority of interventions showed larger effects, but no specific reminder or contextual features were significantly associated with effect magnitude. Further research must identify design features and contextual factors consistently associated with larger improvements in provider behaviour if computer reminders are to succeed on more than a trial and error basis. | 28 randomised controlled trials. Median absolute improvement of care (process adherence) was 4.2 % (IQR +0.8 % to +18.8 %). | Encourage the use of point of care reminders, ideally computer reminders but if not feasible paper reminders such as sticker checklists on patient notes. |
| Educational outreach visits ^6^ | Use of a trained person who meets with providers in their practice settings to give information with the intent of changing the providers’ practice. The information given may have included feedback on the performance of the provider(s). | Target: an individual’s knowledge and attitudes (predominately target prescribing behaviours). Practicalities: considerable resources including the costs of detailers and preparation of materials. | Educational outreach visits alone or when combined with other interventions have effects on prescribing that are relatively consistent and small, but potentially important. Their effects on other types of professional performance vary from small to modest improvements, and it is not possible from this review to explain that variation. | 69 randomised controlled trials involving 15,000 + health professionals. Median adjusted risk difference (RD) in compliance with desired practice was 5.6 % (IQR 3.0 % to 9.0 %). The adjusted RDs were highly consistent for prescribing (median 4.8 %, IQR 3.0 % to 6.5 % for 17 comparisons), but varied for other types of professional performance (median 6.0 %, IQR 3.6 % to 16.0 % for 17 comparisons). EOVs appeared to be slightly superior to audit and feedback | [Note: Although it was found that EOVs were effective, its use in improving prescribing practice was deemed the most consistent result. As prescribing is not included in the target behaviours, its applicability was questioned. The considerable cost of including this component in an intervention that will be implemented in a large number of hospitals, located in diverse locations was also seen as a reason for not including it as an intervention component. |

1. Forsetlund L, Bjorndal A, Rashidian A, Jamtvedt G, O’Brien MA, Wolf F, et al. Continuing education meetings and workshops: effects on professional practice and health care outcomes. Cochrane Database Syst Rev. 2009;3:CD003030.
2. Flodgren G, Parmelli E, Doumit G, Gattellari M, O’Brien MA, Grimshaw J, et al. Local opinion leaders: effects on professional practice and health care outcomes. Cochrane Database Syst Rev. 2011;8:CD000125.
3. Giguere A, Legare F, Grimshaw J, Turcotte S, Fiander M, Grudniewicz A, et al. Printed educational materials: effects on professional practice and healthcare outcomes. Cochrane Database Syst Rev. 2012;10:CD004398.
4. Ivers N, Jamtvedt G, Flottorp S, Young JM, Odgaard-Jensen J, French SD, et al. Audit and feedback: effects on professional practice and healthcare outcomes. Cochrane Database Syst Rev. 2012;6:CD000259.
5. Shojania KG, Jennings A, Mayhew A, Ramsay CR, Eccles MP, Grimshaw J. The effects of on-screen, point of care computer reminders on processes and outcomes of care. Cochrane Database Syst Rev. 2009;3:CD001096.
6. O’Brien MA, Rogers S, Jamtvedt G, Oxman AD, Odgaard-Jensen J, Kristoffersen DT, et al. Educational outreach visits: effects on professional practice and health care outcomes. Cochrane Database Syst Rev. 2007;17:CD000409.
